# Sea Buckthorn Pericarp Flavonoids Improve Diet-Induced Hyperlipidemia via Coordinated Modulation of Hepatic Lipid Metabolism and Gut Microbiota

**DOI:** 10.3390/foods15061049

**Published:** 2026-03-17

**Authors:** Xiaowei Bao, Qin Wang, Fengming Li, Tonghua Wu, Xiaojuan Mou, Qiqi Zeng, Mingxi Jia

**Affiliations:** 1 College of Food Science and Pharmacy, Xinjiang Agricultural University, Urumqi 830052, China; 120050032@xjau.edu.cn (X.B.); 17716982080@163.com (X.M.); 18299158021@163.com (Q.Z.); 2 Karamay Green into Agricultural Development Limited Liability Company, Karamay 834000, China; 1331900918@163.com; 3 Xinjiang Seabuckthorn Deep Processing Engineering Technology Research Center, Akqi County 843500, China; 18338291870@163.com; 4 College of Animal Science, Xinjiang Agricultural University, Urumqi 830052, China; lifming@163.com

**Keywords:** total flavonoids of Sea buckthorn pericarp pomace (TFSP), high-fat diet (HFD), hyperlipidemia, lipid metabolism, intestinal flora

## Abstract

Sea buckthorn pericarp pomace, a major by-product of juice processing, represents a promising food-grade source of bioactive flavonoids. This study investigated the hypolipidemic effects and underlying mechanisms of total flavonoids extracted from Sea buckthorn pericarp pomace (TFSP) in mice with high-fat diet (HFD)-induced hyperlipidemia. TFSP intervention significantly suppressed body weight gain and improved serum lipid profiles by reducing total cholesterol (TC), triglycerides (TG), and low-density lipoprotein cholesterol (LDL-C), while increasing high-density lipoprotein cholesterol (HDL-C). Hepatic lipid accumulation and injury were alleviated, accompanied by enhanced activities of antioxidant enzymes (SOD, CAT, GSH-Px) and reduced oxidative stress markers. At the molecular level, TFSP downregulated key lipogenic proteins—including ACC and FAS—and upregulated markers of fatty acid oxidation and triglyceride hydrolysis—namely CPT-1α, PPARα, and ATGL. Moreover, TFSP restored HFD-induced gut microbiota dysbiosis, increased the relative abundance of beneficial genera such as *Akkermansia*, and decreased that of potentially harmful taxa including *Allobaculum*. These findings demonstrate that TFSP—a value-added food processing by-product—ameliorates hyperlipidemia through coordinated regulation of hepatic lipid metabolism and gut microbial composition, supporting its potential application as a natural, food-derived ingredient in lipid-lowering functional foods.

## 1. Introduction

Sea buckthorn fruit is extensively utilized in juice processing, generating substantial pericarp residue as a major by-product accounting for approximately 20% of the fresh fruit weight. This underutilized food processing residue represents a promising source of bioactive flavonoids and dietary fiber, highlighting its potential for valorization in functional food applications. Flavonoids, including isorhamnetin, quercetin, and kaempferol, are recognized as key bioactive components in Sea buckthorn with demonstrated relevance to food chemistry and nutrition. These compounds not only help regulate lipid metabolism but also enhance the diversity and stability of gut microbiota, contributing to metabolic homeostasis [1]. Recent studies have shown that Sea buckthorn flavonoids can inhibit low-density lipoprotein cholesterol (LDL-C) oxidation and elevate high-density lipoprotein cholesterol (HDL-C) levels, improving blood lipid profiles [2,3]. Additionally, these flavonoids exhibit hypolipidemic effects in high-fat diet (HFD)-induced obese mice and modulate intestinal microbiota composition, further supporting their potential as functional food ingredients [2,4].

Prolonged consumption of a high-fat diet leads to dyslipidemia, oxidative stress imbalance, and gut microbiota dysbiosis, ultimately contributing to hyperlipidemia [5]. From a food chemistry perspective, understanding how dietary components influence metabolic pathways is essential for developing functional foods. Key regulatory proteins involved in lipid metabolism include AMP-activated protein kinase α (AMPKα), acetyl-CoA carboxylase (ACC), fatty acid synthase (FAS), carnitine palmitoyltransferase-1α (CPT-1α), and adipose triglyceride lipase (ATGL) [6,7]. These enzymes and signaling molecules collectively regulate lipid synthesis, transport, and catabolism. Peroxisome proliferator-activated receptor alpha (PPARα) serves as a crucial nuclear receptor regulating fatty acid oxidation, triacylglycerol synthesis, and cholesterol metabolism [7]. Under HFD conditions, reactive oxygen species (ROS) levels in the liver increase significantly, leading to hepatic injury, while endogenous antioxidant enzymes including superoxide dismutase (SOD), catalase (CAT), and glutathione peroxidase (GSH-Px) play vital roles in mitigating oxidative damage [8].

Furthermore, HFD consumption alters intestinal microbiota composition and function, affecting microbial diversity, abundance, and metabolic activity [9,10]. HFD-induced intestinal oxidative stress and increased permeability create a vicious cycle that damages the intestinal barrier, allowing endotoxin translocation into circulation and triggering systemic inflammation [11,12]. These changes further exacerbate hyperlipidemia and modify the gut microbial ecosystem.

In our previous study, we successfully extracted total flavonoids from Sea buckthorn pericarp pomace (TFSP) with a purity of 50.204% [13]. Building on this work, the current study establishes a HFD-induced hyperlipidemia model in C57BL/6J mice to comprehensively evaluate the effects of TFSP on body weight, serum parameters, liver biochemistry, histopathological features, lipid metabolic pathways, and intestinal microbiota composition. This investigation aims to elucidate the efficacy and mechanisms through which TFSP improves hepatic lipid metabolism and intestinal microbiota in hyperlipidemic mice, providing a scientific foundation for developing Sea buckthorn pericarp pomace as a hypolipidemic functional food ingredient.

## 2. Materials and Methods

### 2.1. Materials

Sea buckthorn pericarp residue was supplied by Huihua Biotechnology Co., Ltd. (Altay, Xinjiang, China). The mice’s high-fat diet, consisting of 20% protein, 40% carbohydrate, and 40% fat, was provided by Xietong Biological Co., Ltd. (Jiangsu, China). The lipid-lowering drug, Xuezhikang (XZK), was sourced from Beida Weixin Biotechnology Co., Ltd. (Beijing, China, Cat. No. 210060032). Total cholesterol (TC) kit (Cat. No. A111-1), triglycerides (TG) kit (Cat. No. A110-1), high-density lipoprotein (HDL-C) kit (Cat. No. A112-1), low-density lipoprotein (LDL-C) kit (Cat. No. A113-1), aspartate aminotransferase (AST) kit (Cat. No. C010-2), alanine aminotransferase (ALT) kit (Cat. No. C009-2), alkaline phosphatase (ALP) kit (Cat. No. A059-2), lactate dehydrogenase (LDH) kit (Cat. No. A020-2), superoxide dismutase (SOD) kit (Cat. No. A001-1), catalase (CAT) kit (Cat. No. A007-2), glutathione peroxidase (GSH-PX) kit (Cat. No. A005), and malondialdehyde (MDA) (Cat. No. A003-1) kits were procured from Nanjing Jiancheng Bioengineering Institute (Jiangsu, China). Peroxisome proliferator-activated receptor alpha (PPARα) primary antibody (Cat. No. A00600-2), carnitine palmitoyltransferase-1 alpha (CPT-1α) primary antibody (Cat. No. A00917-3), acetyl-CoA carboxylase (ACC) primary antibody (Cat. No. M01802-2), fatty acid synthase (FAS) primary antibody (Cat. No. BA0484), adipose triglyceride lipase (ATGL) primary antibody (Cat. No. A01800-1), and GAPDH primary antibody (Cat. No. BM1623) were obtained from BOSTER Biological Technology Co., Ltd. (Wuhan, China).

### 2.2. TFSP Extraction and Ultra Performance Liquid Chromatography–Quadrupole Mass Spectrometry (UPLC-Q-MS) Assay

The TFSP were extracted according to our previous method [13]. Briefly, Sea buckthorn pericarp pomace was passed through a 40-mesh sieve, and 2.0 g was accurately weighed. Ultrasonic-assisted extraction was performed using 59% (*v*/*v*) ethanol at a liquid-to-solid ratio of 40 mL/g, temperature of 59 °C, and an extraction time of 39 min. The extraction was repeated twice, and the combined extracts were freeze-dried to a paste-like consistency. Further purification was carried out using AB-8 macroporous resin. A sample solution at a concentration of 2.2 mg/mL (pH 5) was loaded onto the resin column at a flow rate of 1.0 mL/min with a loading volume of 140 mL. Elution was performed using 75% ethanol at a flow rate of 1.5 mL/min with an elution volume of 120 mL. The eluate was freeze-dried, and the resulting product was designated as TFSP. The total flavonoid content was determined using the aluminum chloride colorimetric method [14], revealing an increase in purity from 9.78% before purification to 50.20% after purification. Further, the specific composition and content of each substance in TFSP was detected by UPLC-Q-MS. For UPLC-Q-MS analysis, we specify the column (Waters HSS T3, 50 mm × 2.1 mm, 1.8 µm) maintained at 40 °C. The mobile phase consisted of ultrapure water containing 0.1% formic acid (A) and acetonitrile containing 0.1% formic acid (B) at a flow rate of 0.3 mL/min. The gradient elution program was as follows: 0–2 min, 90% A (10% B); 2–6 min, 90–40% A (10–60% B); 6–9 min, 40% A (60% B); 9–9.1 min, 40–90% A (60–10% B); 9.1–12 min, 90% A (10% B). The injection volume was 2 µL. Mass spectrometry was conducted in negative ion mode using selected ion monitoring (SIM) with a full scan range of m/z 100–900. The ion spray voltage was set at −2800 V, sheath gas at 40 arb, auxiliary gas at 10 arb, ion transfer tube temperature at 320 °C, and vaporizer temperature at 350 °C. A series of mixed standard working solutions at different concentrations were prepared by diluting the mixed standard stock solution according to the concentration range required for calibration. Calibration curves were constructed using reference standards over appropriate concentration ranges; all compounds showed good linearity (R^2^ > 0.999). The limits of detection and quantification were determined based on signal-to-noise ratios of 3 and 10, respectively. Intra-day and inter-day precision were below 5%, and recovery rates ranged from 95% to 105%, confirming the reliability of the method. Each sample was analyzed in triplicate to ensure reproducibility.

### 2.3. Establishment of Animal Models and Group Processing

SPF-grade male C57BL/6J mice, weighing 18 ± 2 g, were utilized for the experiments. These animals were procured from Hunan Slake Jinda Laboratory Animal Co., Ltd. (Changsha, China. License No. SCXK 2019-0004). All experimental protocols involving animals were reviewed and approved by the Experimental Animal Welfare Ethics Committee of Xinjiang Agricultural University (Approval No. 2023026). Forty male mice were randomly allocated into 5 groups, each comprising 8 animals. The normal control (NC) group was fed a standard chow diet, while the other 4 groups were provided with a high-fat diet for 30 consecutive days to induce hyperlipidemia. Subsequently, the mice were subjected to drug gavage for 30 d, during which their body weights were weighed every 6 d and recorded. The doses administered to mice were calculated by converting the clinically recommended human doses. The groups included: NC and model (HFD) groups receiving 0.9% saline at 0.2 mL/day, the positive control group receiving Xuezhikang (XZK) at 180 mg/kg/day, the low-dose group receiving TFSP (L-TFSP) at 160 mg/kg/day, and the high-dose group receiving TFSP (H-TFSP) at 320 mg/kg/day [15].

### 2.4. Determination of Serum Parameters in Mice

Mice were fasted for 12 h prior to blood collection. Blood samples were obtained via orbital puncture and left at 4 °C for 2 h. Serum was subsequently isolated by centrifugation at 2000 rpm for 10 min. The serum concentrations of TC, TG, HDL-C, LDL-C, AST, ALT, AKP and LDH were measured following the manufacturer’s protocol. Each assay was calibrated using the standards provided in the respective kits, and a standard curve with a correlation coefficient (R^2^) greater than 0.99 was required for acceptance. Blank samples (reagent only) and quality control (QC) samples (supplied with the kits) were included in each run to monitor background and assay performance. All samples were measured in duplicate, and the mean values were used for statistical analysis. The coefficients of variation (CV) for both intra-assay and inter-assay were maintained below 10% for all parameters, confirming the reproducibility of the assays.

### 2.5. Determination of Liver Parameters

The livers were isolated from dissected mice and thoroughly rinsed with saline buffer. An appropriate amount of liver tissue was weighed and added to ice-cold sterile saline at a ratio of 1:9 (*w*/*v*) for homogenization. The homogenate was centrifuged at 4 °C, 2500 r/min for 10 min, and the supernatant was collected. The concentrations of TC, TG, HDL-C, LDL-C, SOD, GSH-PX, CAT and MDA in the liver homogenate were measured according to the manufacturer’s instructions. Each assay was calibrated with standards provided in the kits, and blank, standard, and quality control samples were run alongside experimental samples. All measurements were performed in duplicate, and the mean values were used for statistical analysis. Intra-assay and inter-assay coefficients of variation (CV) were maintained below 10%, respectively, to ensure consistency.

### 2.6. Histopathological Tests

Tissue samples were fixed in 10% neutral buffered paraformaldehyde, dehydrated and hyalinized. They were subsequently embedded in paraffin, and 5 μm sections were prepared. Hematoxylin–eosin (H&E) staining was performed according to standard protocols, and the slides were examined under a microscope and photographed for documentation.

### 2.7. Western Blot

Weigh 0.1~0.2 g of liver tissue samples, add RIPA lysis buffer at a ratio of 1:10 (*w*/*v*), and homogenize the samples on ice. Following this, incubate the homogenate at 4 °C for 30 min, then centrifuge at 10,000 rpm for 10 min at 4 °C. Collect the supernatant to obtain the liver tissue protein extract. Protein concentration was determined using the BCA method (Beyotime, Shanghai, China) with bovine serum albumin (BSA) as the standard. Equal amounts (30 µg) were separated by SDS-PAGE and transferred to polyvinylidene difluoride (PVDF, 0.45 μm) membrane. Subsequently, the procedure was performed following the established protocol [16]: blocking with 5% (*w*/*v*) BSA in TBST for 1 h at room temperature; overnight incubation at 4 °C with primary antibody diluted to the manufacturer’s recommended concentration in blocking buffer; and subsequent 1 h incubation at room temperature with HRP-conjugated secondary antibody, also diluted in blocking buffer. All experiments were performed in triplicate. Band intensities were quantified using ImageJ 1.8.0 software with GAPDH as internal control, and fold changes relative to control were calculated.

### 2.8. Mice Fecal DNA Extraction and High-Throughput Sequencing

Mice intestinal contents were collected in sterilized tubes under aseptic conditions. Total DNA was extracted using a DNA extraction kit, and the purity and concentration were assessed by 1% agarose gel electrophoresis and NanoDrop One spectrophotometry (Thermo Fisher Scientific, Wilmington, DE, United States). Subsequently, the V3–V4 hypervariable region of the 16S rRNA gene was amplified using specific forward (5′-ACTCCTACGGGAGGCAGCA-3′) and reverse (5′-GGACTACHVGGGTWTCTAAT-3′) primers. DNA libraries were constructed using the NEB Next^➅^ Ultra™ II FS DNA PCR-free Library Prep Kit (New England Biolabs, Massachusetts, United States). PE250 (double-end sequencing 250 bp) sequencing and analysis was performed by Biomarker Technologies (Beijing, China) using the illumina NovaSeq 6000 platform (Illumina, San Diego, CA, USA).

### 2.9. Statistical Analysis

All experimental data are presented as mean ± standard deviation (SD) and analyzed using SPSS 25.0 statistical software. The Shapiro–Wilk test was performed to assess data normality, and results indicated that all continuous quantitative variables were normally distributed (*p* > 0.05). Given the balanced sample size (*n* = 6 per group), one-way ANOVA—which was used for multi-group comparisons—remained robust to minor deviations from normality; pairwise comparisons were conducted using the LSD test, which maintains adequate statistical power under balanced designs. Additionally, for gut microbiota analyses, principal component analysis (PCA) was performed to visualize overall structural shifts in microbial communities across groups. Prior to PCA, genus-level relative abundance data were centered and scaled to unit variance to ensure an equal contribution of each variable; PCA was implemented using the prcomp function in R (version 4.1.0) with the vegan package (version 2.6-4). Permutational multivariate analysis of variance (PERMANOVA), based on Bray–Curtis dissimilarity and using 999 permutations, was employed to test for significant differences in microbial community composition among groups. *p* < 0.05 was considered statistically significant, and exact *p*-values are reported for all statistical tests in the main text and figure legends.

## 3. Results

### 3.1. Flavonoid Content in TFSP

**Figure 1:**
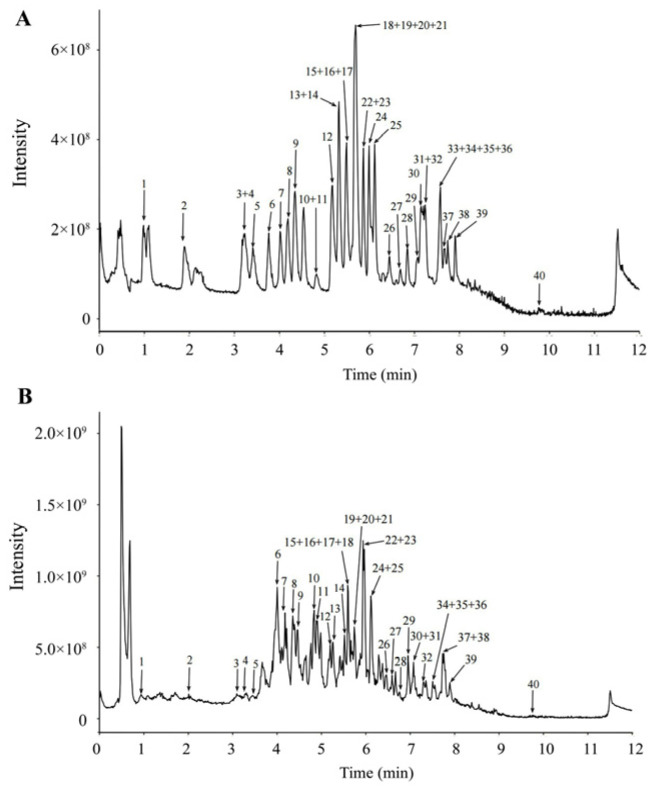
UPLC-Q-MS in negative ion mode (**A**) mixed standard and (**B**) TFSP trace particle flow diagram (TIC).

The components of TFSP were quantified using UPLC-Q-MS, and a total of 20 flavonoids, two polyphenols and 17 phenolic acids were identified (Figure 1 and Table 1). All substances were successfully detected except for Naringenin Chalcone, which remained undetected. Among the detected compounds in TFSP, Rutin was found to be the most abundant, with a concentration of 1348.811 ± 0.179 ng/mg. This was followed by Catechin (994.812 ± 0.845 ng/mg), Quercetin 3-β-D-glucoside (558.638 ± 0.391 ng/mg), Kaempferol-3-O-glucoside (102.69 ± 0.427 ng/mg), Isorhamnetin (182.445 ± 0.62 ng/mg) and Quercetin (111.289 ± 0.254 ng/mg). It was demonstrated that flavonoids and phenolic acids in Sea buckthorn pericarp pomace could be efficiently extracted using AB-8 macroporous resin purification, contributing positively to the effective utilization of Sea buckthorn by-products. The TFSP contains trace amounts of potentially toxic bioactive components, and some of these components may pose safety concerns at high doses. Taking gossypol (Peak 40 in Table 1) as a representative example, this polyphenolic compound is toxic at high concentrations. The gossypol content detected in TFSP is extremely low (0.344 ± 0.024 ng/mg, equivalent to 0.344 µg/g). It has been reported that the no-observed-adverse-effect level (NOAEL) of gossypol for rodents is >100 mg/kg body weight/day [17]. In this study, the maximum TFSP dose given to mice was 320 mg/kg/day, corresponding to an intake of approximately 0.11 µg/kg/day of gossypol—far below the toxicity threshold. Therefore, the TFSP dosage used in this study exhibits a high degree of safety.

### 3.2. TFSP Ameliorates Weight Gain and Body Fat Accumulation in HFD Mice

**Figure 2:**
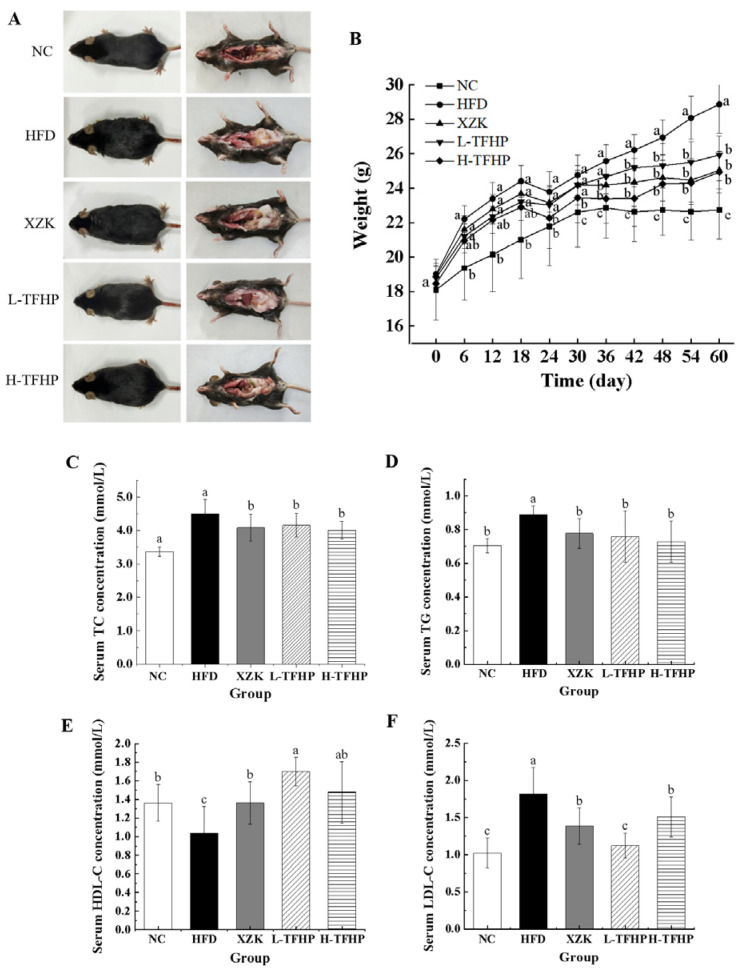
TFSP improves the physical status of HFD mice. (**A**) Mice body map and abdominal anatomy; (**B**) longitudinal tracking of mice body weight, *n* = 8; (**C**) TC concentration in serum, *n* = 6; (**D**) TG concentration in serum, *n* = 6; (**E**) HDL-C concentration in serum, *n* = 6; (**F**) LDL-C concentration in serum, *n* = 6. Data are presented as mean ± SD. Different letters above bars indicate statistically significant differences (*p* < 0.05) by one-way ANOVA followed by LSD post hoc test.

As illustrated in Figure 2A, compared to the NC group, mice in the HFD group exhibited obesity. In contrast, the experimental group demonstrated a progressive improvement in body morphology as the administered dose increased. Abdominal anatomical drawings revealed substantial accumulation of subcutaneous abdominal fat in the HFD group of mice. In comparison to the HFD group, there was a notable reduction in subcutaneous abdominal fat accumulation in the XZK, L-TFSP, and H-TFSP groups. During the 60-day experimental period, all groups of mice exhibited varying degrees of body weight gain (Figure 2B). By day 30 of the experiment, the body weights of mice in both the HFD and experimental groups were significantly higher compared to those in the NC group (*p* < 0.05). On the 60th day, the body weight of mice in the HFD group increased by 26.91%, significantly higher than that of the NC group (*p* = 0.0084). In comparison, the body weights of mice in the XZK, L-TFSP, and H-TFSP groups were markedly lower than those in the HFD group, with reductions of 13.24% (*p* = 0.0153), 10.19% (*p* = 0.0186), and 13.62% (*p* = 0.0084), respectively.

The effects of TFSP intervention on serum concentrations of TC, TG, LDL-C and HDL-C in hyperlipidemic mice are illustrated in Figure 2C–F. Compared with the NC group, mice in the HFD group exhibited significantly elevated serum concentrations of TC (*p* = 0.0103), TG (*p* = 0.0314) and LDL-C (*p* = 0.0097), along with markedly reduced concentrations of HDL-C (*p* = 0.0386). Compared with the HFD group, the serum TC levels in the XZK, L-TFSP, and H-TFSP groups decreased by 9.37% (*p* = 0.0415), 7.75% (*p* = 0.0402) and 11.05% (*p* = 0.0374), respectively; TG levels decreased by 12.43% (*p* = 0.0217), 14.50% (*p* = 0.0435) and 17.89% (*p* = 0.0382), respectively; LDL-C levels decreased by 23.75% (*p* = 0.0195), 38.24% (*p* = 0.0125) and 17.14% (*p* = 0.0302), respectively; and HDL-C levels increased by 31.52% (*p* = 0.0215), 63.76% (*p* = 0.0103) and 42.36% (*p* = 0.0387), respectively. These results indicated that TFSP can effectively regulate blood lipid profiles in mice.

### 3.3. TFSP Improves Liver Fat Accumulation in Mice

**Figure 3:**
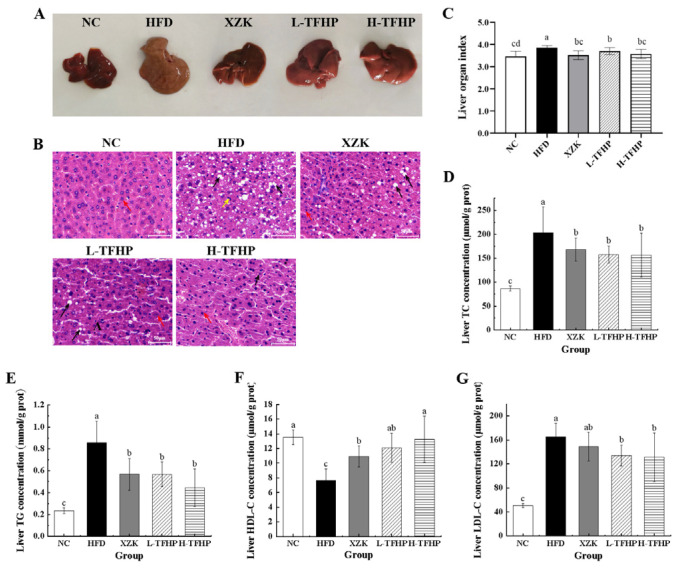
TFSP improves liver fat accumulation in mice. (**A**) Appearance of liver organs in mice; (**B**) H&E staining of liver tissue (magnification: 10 × 40); (**C**) liver coefficients; (**D**) TC content in liver tissue; (**E**) TG content in liver tissue; (**F**) HDL-C content in liver tissue; (**G**) LDL-C content in liver tissue. *n* = 6 per group. Data are presented as mean ± SD. Different letters above bars indicate statistically significant differences (*p* < 0.05) by one-way ANOVA followed by LSD post hoc test.

Morphological observations of the livers revealed that those in the NC group exhibited regular morphology, a bright red color, smooth texture, and appropriate elasticity (Figure 3A). In contrast, the livers in the HFD group were notably enlarged, with a brownish-yellow hue, greasy consistency, poor elasticity, and a mottled surface. Compared to the HFD group, the livers in the XZK, L-TFSP and H-TFSP groups showed less yellowing and greasiness, with a reduction in yellow spots on the surface. H&E staining of liver tissues from mice (Figure 3B) revealed that, compared with the NC group, the HFD group exhibited markedly abnormal liver architecture. Specifically, hepatocytes in the HFD group displayed extensive swelling and diffuse steatosis, disorganized hepatic cords, cytoplasmic vacuoles of varying sizes (black arrowheads), and displaced nuclei. This phenomenon was significantly alleviated by both the positive drug and TFSP interventions. In the XZK group, liver tissue exhibited mild structural abnormalities, characterized by reduced cellular swelling and fewer rounded fat vacuoles (black arrows), with a greater proportion of hepatocytes appearing normal (red arrows). The L-TFSP group exhibited mild hepatocellular steatosis, characterized by fewer lipid droplets and relatively preserved cell morphology. In contrast, the H-TFSP group demonstrated the most significant treatment effect, with nearly normal liver tissues, well-organized cellular structures, and minimal fat vacuoles (black arrows). Figure 3C demonstrates that the liver organ coefficient in the HFD group was significantly higher (*p* = 0.0473) compared to the NC group, suggesting that the high-fat diet resulted in increased hepatic fat accumulation in mice. Conversely, the liver organ coefficients in the XZK (*p* = 0.0395), L-TFSP (*p* = 0.0488) and H-TFSP (*p* = 0.0403) groups were markedly lower than those in the HFD group.

The liver serves as the principal organ for lipid synthesis and metabolism. Excessive accumulation of lipids within hepatocytes can result in fatty liver formation, which subsequently elicits a cascade of inflammatory responses and may ultimately culminate in irreversible hepatic damage. Compared with the NC group, the HFD significantly increased hepatic TC, TG and LDL-C concentrations while markedly decreasing HDL-C concentration in mice in the HFD group (Figure 3D–G). TFSP treatment significantly ameliorated the HFD-induced alterations in hepatic lipid profiles. Specifically, compared to the HFD group, the livers of mice in the H-TFSP group exhibited the most substantial reductions in TC, TG and LDL-C concentrations, by 23.14% (*p* = 0.0386), 47.98% (*p* = 0.0162) and 20.54% (*p* = 0.0314), respectively. Additionally, HDL-C concentration was significantly elevated by 73.14% (*p* = 0.0116). These findings suggested that TFSP can effectively protect mouse liver tissue and mitigate hepatic fat accumulation.

### 3.4. TFSP Ameliorates HFD-Induced Hepatic Impairment in Mice

**Figure 4:**
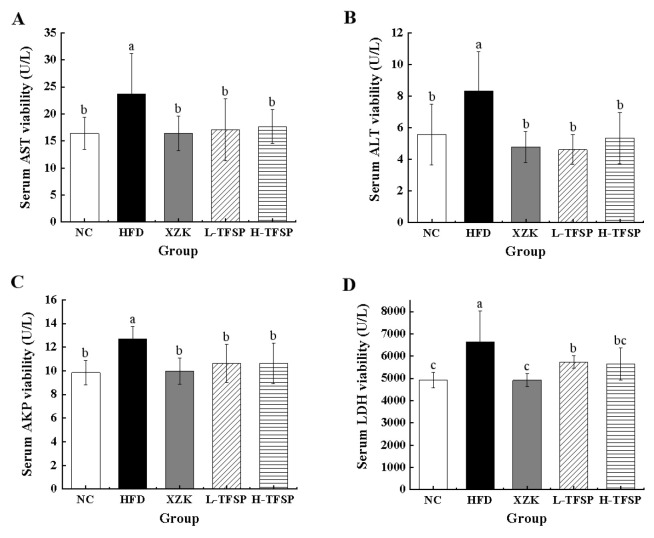
Biomarkers of hepatic impairment in serum. (**A**) AST; (**B**) ALT; (**C**) AKP; (**D**) LDH. *n* = 6 per group. Data are presented as mean ± SD. Different letters above bars indicate statistically significant differences (*p* < 0.05) by one-way ANOVA followed by LSD post hoc test.

AST, ALT, ALP and LDH are critical biochemical markers for evaluating liver function. Upon liver tissue injury, these substances are released into the bloodstream in significant amounts, allowing the degree of liver damage to be assessed through serum level measurements of these indicators. As illustrated in Figure 4, all four serum liver function indices were markedly elevated in the HFD group relative to the NC group (*p* < 0.05), suggesting that a high-fat diet can induce hepatic injury. Compared with the HFD group, the levels of AST, ALT, ALP and LDH were significantly reduced in the XZK, L-TFSP and H-TFSP groups (*p* < 0.05). Among them, the effects of the XZK and L-TFSP groups on reducing AST and ALT levels were more pronounced (*p* < 0.05), while the XZK and H-TFSP groups showed significant effects in decreasing AKP and LDH levels (*p* < 0.05).

**Figure 5:**
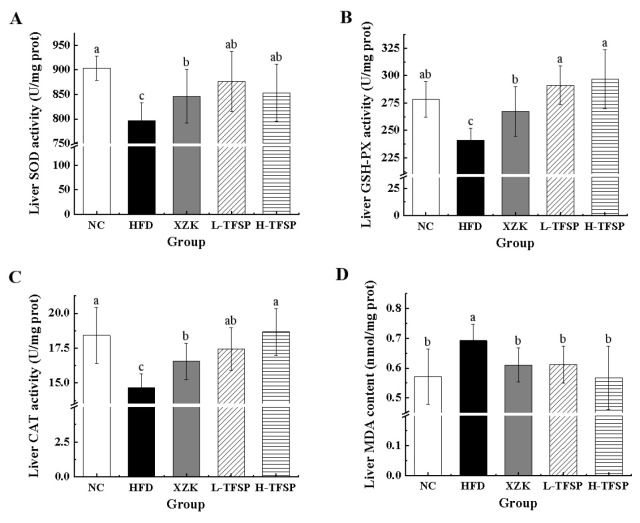
Effect of TFSP on the activity of endogenous antioxidant enzyme system in the liver of HFD mice. (**A**) SOD; (**B**) GSH-PX; (**C**) CAT; (**D**) MDA. *n* = 6 per group. Data are presented as mean ± SD. Different letters above bars indicate statistically significant differences (*p* < 0.05) by one-way ANOVA followed by LSD post hoc test.

HFD-induced hyperlipidemia not only enhances inflammatory responses and lipid oxidative stress in the liver but also inhibits the activity of endogenous antioxidant enzyme systems, thereby exacerbating liver injury. The activities of SOD (*p* = 0.0146), GSH-Px (*p* = 0.0167) and CAT (*p* = 0.0314) in the liver tissues of the HFD group were significantly lower compared to those in the NC group, while the MDA content was significantly higher (*p* = 0.0357, Figure 5). Compared to the HFD group, the antioxidant enzyme activities were significantly enhanced in the XZK, L-TFSP and H-TFSP groups. Specifically, the SOD activity increased by 10.07% (*p* = 0.0374) in the L-TFSP group, whereas the activities of GSH-Px and CAT increased by 23.08% (*p* = 0.0142) and 27.10% (*p* = 0.0175), respectively, in the H-TFSP group. Additionally, the MDA content decreased by 18.05% (*p* = 0.0226) in the H-TFSP group. These results demonstrated that TFSP was effective in reducing blood lipid levels while markedly enhancing liver function.

### 3.5. TFSP Regulates the Relevant Pathways of Lipid Metabolism

**Figure 6:**
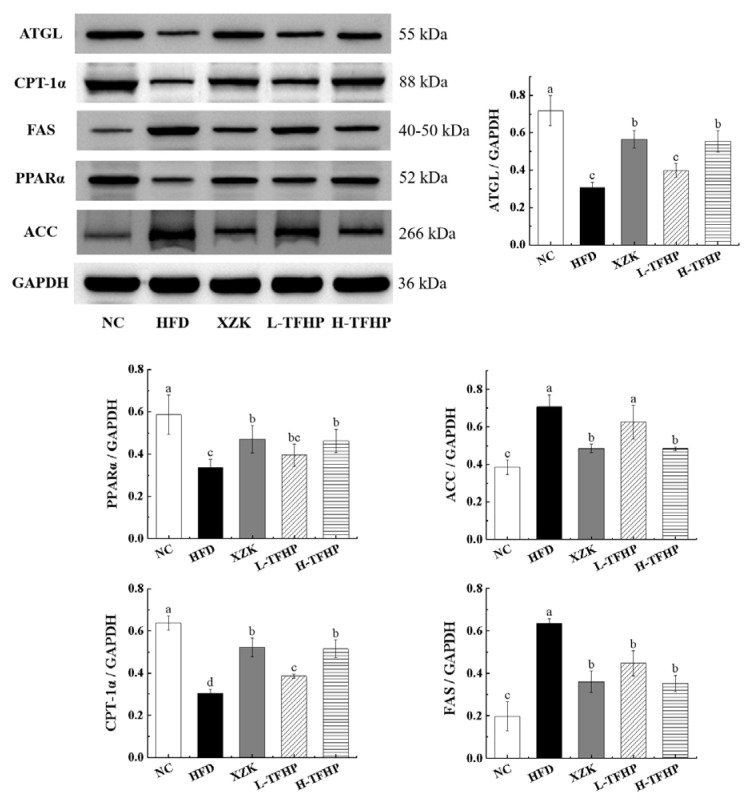
TFSP modulates the expression of ACC, FAS, CPT-1α, PPARα and ATGL proteins in the livers of HFD mice. *n* = 6 per group. Data are presented as mean ± SD. Different letters above bars indicate statistically significant differences (*p* < 0.05) by one-way ANOVA followed by LSD post hoc test.

It has been demonstrated that ACC and FAS are involved in de novo lipid synthesis, PPARα and CPT-1α play crucial roles in mitochondrial fatty acid oxidation, while ATGL mediates the hydrolysis of triglycerides. To further elucidate the regulatory effects of TFSP on lipid metabolism, we investigated the expression levels of these proteins in the livers of different groups of mice (Figure 6). Compared to the NC group, the HFD group significantly upregulated the protein expression of ACC (*p* = 0.00823) and FAS (*p* = 0.00754) while inhibiting the expression of CPT-1α (*p* = 0.00113), PPARα (*p* = 0.00967) and ATGL (*p* = 0.00658). In comparison with the HFD group, the L-TFSP group exhibited reduced protein expression of FAS (*p* = 0.0275) but increased expression of CPT-1α (*p* = 0.0387). The H-TFSP group showed a more pronounced effect, characterized by downregulation of ACC (*p* = 0.0164) and FAS (*p* = 0.0115) expression along with upregulation of CPT-1α (*p* = 0.00873), PPARα (*p* = 0.0235) and ATGL (*p* = 0.0182) expression. These results suggested that TFSP may influence lipid metabolism regulation through the downregulation of ACC and FAS, alongside the upregulation of CPT-1α, PPARα and ATGL.

### 3.6. TFSP Improves Gut Flora Diversity

**Figure 7:**
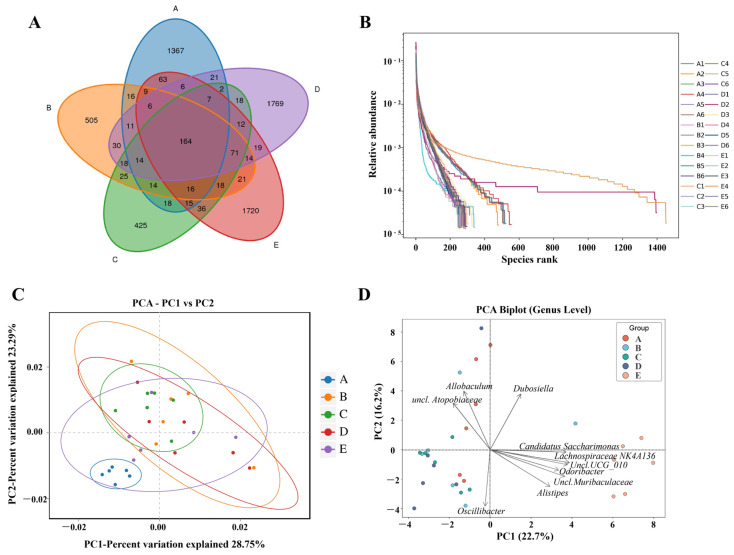
Diversity analysis of intestinal flora. (**A**) Venn diagram; (**B**) rank abundance curves; (**C**) PCA; (**D**) PCA loading (vector) plot. Note: Group A served as the NC group, Group B as the HFD group, Group C as the XZK treatment group, Group D as the L-TFHP group, and Group E as the H-TFHP group; each group comprised six animals (*n* = 6).

Furthermore, we evaluated the regulatory effects of TFSP on the intestinal microbiota of HFD mice. As illustrated in Figure 7A, compared to the NC group, the number of features specific to the HFD group was downregulated. In contrast, both the L-TFSP and H-TFSP groups exhibited a significant increase in the number of these specific features relative to the HFD group (*p* < 0.05). The rank abundance curve showed that the NC group has the widest width on the horizontal coordinate in general, indicating a higher number of species (Figure 7B). The curve of the XZK group, L-TFSP group and H-TFSP group was flat, indicating good species uniformity.

PCA revealed that the first two principal components (PC1 and PC2) explained 28.75% and 23.29% of the total variance, respectively (Figure 7C). The HFD group was distinctly separated from the NC group along the PC1 axis, indicating a substantial alteration in the composition of the intestinal flora due to the HFD (PERMANOVA, *p* = 0.0248). Notably, the H-TFSP-treated group was clearly separated from all other groups along PC1, primarily driven by higher relative abundances of *Lachnospiraceae_NK4A136_group*, *Candidatus_Saccharimonas*, *unclassified Muribaculaceae*, and *Odoribacter*, as shown in the loading plot (Figure 7D). Along the PC2 axis, genera including *Allobaculum*, *Dubosiella*, and *Oscillibacter* were the major contributors to the differentiation among the remaining groups (NC, HFD, XZK, and L-TFSP). The H-TFSP-treated groups were distinctly segregated from the HFD group along both PC1 and PC2 axes, suggesting a significant restoration of the intestinal microbiota composition (PERMANOVA, *p* = 0.0397). To further characterize the gut microbiota alterations, we assessed α-diversity and additional β-diversity metrics. As shown in Figure S1, the HFD group exhibited significantly reduced Ace, Chao1, and Shannon indices compared to the NC group (*p* < 0.05), indicating decreased microbial richness and diversity, while TFSP intervention partially restored these parameters. Complementary β-diversity analyses using Non-metric Multidimensional Scaling (NMDS) confirmed the distinct clustering observed in PCA (Figure S2A; Stress = 0.1222), and ANOSIM verified that between-group differences exceeded within-group variation (Figure S2B; *p* = 0.001). These results indicate that TFSP intervention may enhance the richness and diversity of intestinal microbiota under HFD conditions, thereby potentially contributing to the improvement of intestinal microecology.

### 3.7. Regulation of Intestinal Flora at the Phylum and Genus Level in HFD Mice by TFSP

**Figure 8:**
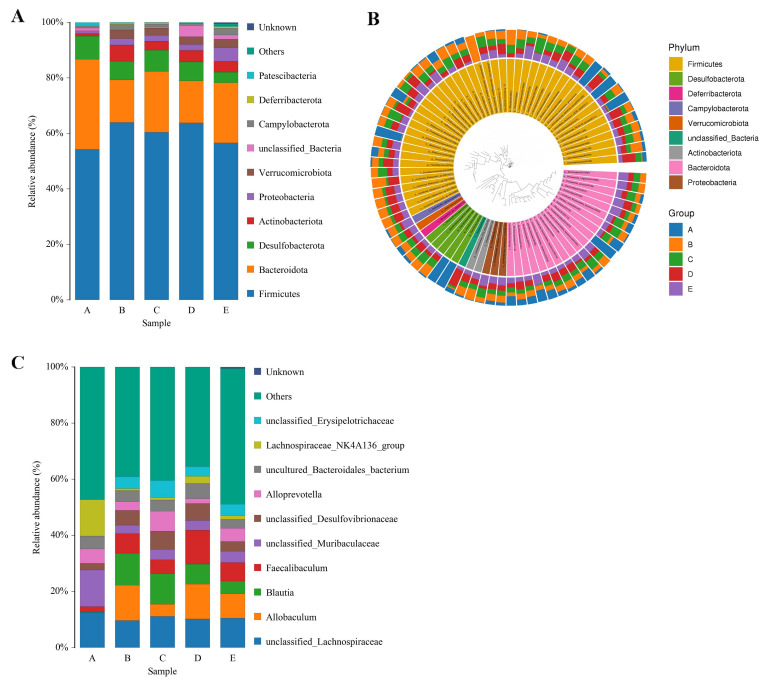
Abundance of gut flora at the phylum and genus level. (**A**) Relative abundance at the gate level; (**B**) circle plots of the top 50 subgroups of ASV abundance; (**C**) relative abundance at genus level. *n* = 6.

Analysis of the gut microbial composition at the phylum level (Figure 8A) revealed minimal variation in community structure. The relative abundance data indicated that *Firmicutes* and *Bacteroidota* were the predominant phyla, with *Bacteroidota* consistently comprising over 50% of the total relative abundance (*p* < 0.05). The top 50 most abundant microorganisms by ASV were predominantly strains of *Firmicutes*, followed by *Bacteroidota*, consistent with the species cumulative distribution histogram at the phylum level (Figure 8B). Compared to the HFD group, the XZK and H-TFSP groups exhibited a decrease in the relative abundance of *Firmicutes* and an increase in the relative abundance of *Bacteroidota*. Notably, the H-TFSP group also showed a significant reduction in the relative abundance of *Desulfobacterota* (*p* < 0.05).

The top ten genera in abundance exhibited notable species variation at the genus level (Figure 8C). Specifically, the relative abundances of *Allobaculum*, *Blautia* and *Faecalibaculum* were significantly higher in the HFD group compared to the NC group, suggesting that these three genera are particularly responsive to a HFD. Compared with the HFD group, the XZK group exhibited a notable reduction in the relative abundance of *Allobaculum* and *Faecalibaculum* (*p* < 0.05); similarly, the TFSP group showed a significant decrease in the relative abundance of *Allobaculum* and *Blautia* compared with the HFD group (*p* < 0.05). These findings suggested that TFSP can effectively alleviate the detrimental effects of a HFD on gut microbiota.

### 3.8. Analysis of Microbial Community Differences

**Figure 9:**
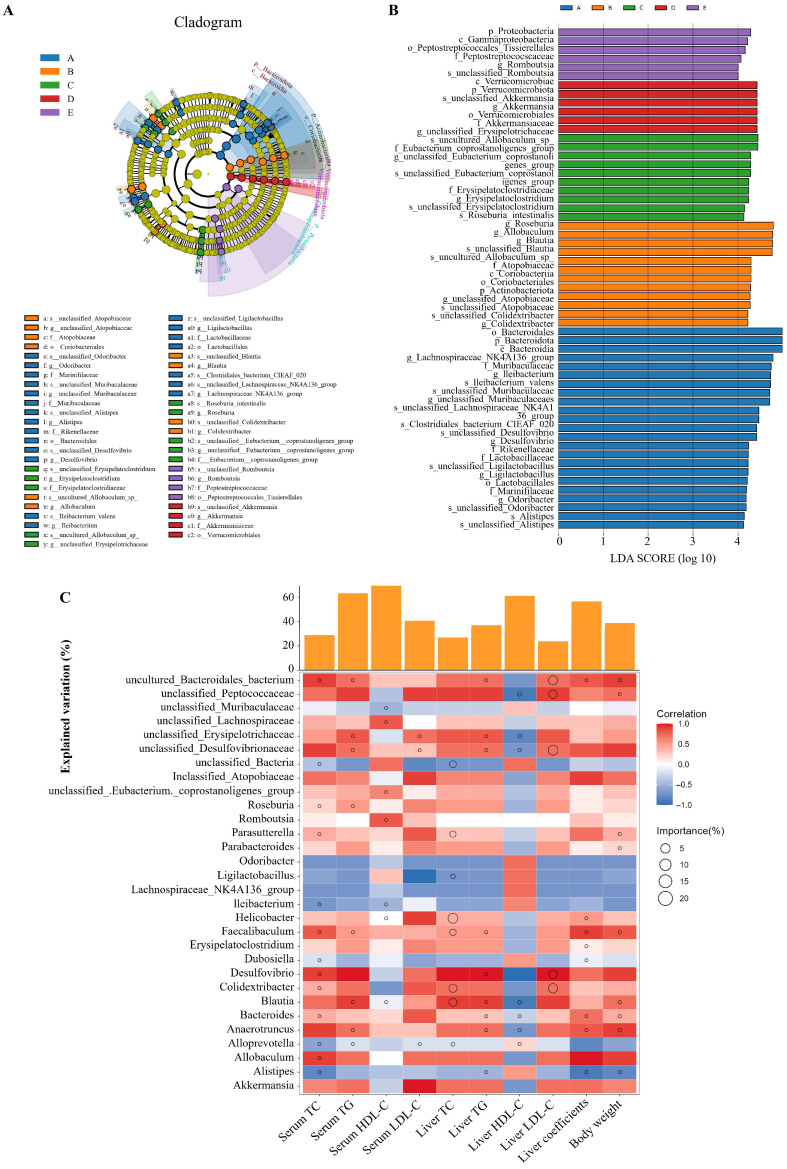
Correlation between intestinal flora and hyperlipidemia. (**A**) Cladogram of the results of LEfSe analyses; (**B**) biomarker screening between different treatment groups based on LEfSe (LDA score > 4); (**C**) correlation analysis of gut microbes at genus level with indicators of hyperlipidemia (*n* = 6).

By employing LEfSe analysis, we identified a substantial number of differentially significant taxa across five sample groups (Figure 9A,B). Significant alterations in the abundance of *Verrucomicrobiota*, *Akkermansia*, *Proteobacteria* and *Romboutsia* were observed in both the H-TFSP and L-TFSP group treatments (*p* < 0.05). *Verrucomicrobiota* inhabit the inner layers of the intestinal mucosa, where they synthesize short-chain fatty acids including propionic acid and butyric acid. *Akkermansia*, a probiotic residing in the gut, mitigates lipid metabolic disorders induced by high-fat diets. *Romboutsia* enhances insulin levels in biological serum and reduces blood glucose levels, thereby regulating glucolipid metabolism. These findings suggested that TFSP can enhance the abundance of beneficial intestinal flora under HFD conditions, thereby improving intestinal health.

### 3.9. Correlation Between Intestinal Flora and Hyperlipidemia

To comprehensively analyze the relationship between gut flora and biochemical markers associated with hyperlipidemia, this study conducted correlation and random forest significance analyses using the top 30 species based on relative abundance. As illustrated in Figure 9C, serum and liver levels of TC, TG, and LDL-C exhibited strong correlations with the abundance of gut microbial communities at the genus level (*p* < 0.05). *Allobaculum* exhibited significant positive correlations with serum TC, liver coefficients, and body weight, while showing significant negative correlations with hepatic HDL-C (*p* < 0.05). In the random forest model, *Allobaculum* had a strong importance for predicting serum TC. *Blautia* and *Desulfovibrio* demonstrated significant positive correlations with serum TG, hepatic TC, TG and LDL-C (*p* < 0.05), as well as significant negative correlations with hepatic HDL-C (*p* < 0.05). Notably, *Blautia* showed strong importance in the random forest model for predicting serum TG, HDL-C, hepatic TC, TG, HDL-C, and body weight. *Desulfovibrio* exhibited strong importance for serum TC, liver TG and LDL-C in the random forest model. *Faecalibaculum* showed a significant positive correlation with liver coefficients (*p* < 0.05) and was identified as highly important for serum TC and TG, liver TC and TG, liver coefficients and body weight in the random forest model. *Odoribacter* spp., *Ligilactobacillus* spp., and *Lachnospiraceae_NK4A136_group* spp. demonstrated a significant positive correlation with liver HDL-C (*p* < 0.05). These findings indicated that gut microbiota may play a crucial role in the hypolipidemic effects of TFSP.

## 4. Discussion

Imbalances in dietary lipid intake and endogenous metabolic regulation can lead to significant alterations in blood lipid parameters. In the context of food science, natural bioactive components that modulate lipid homeostasis are of considerable interest. The present study showed that TFSP intervention was associated with a significant attenuation of HFD-induced body weight gain and an improvement in dyslipidemia. Specifically, TFSP intervention markedly decreased serum and hepatic levels of TC, TG, and LDL-C, while concurrently increasing HDL-C levels [18,19]. These findings highlight the potential of TFSP as a food-derived ingredient with lipid-regulating functions, supporting its prospective application in functional foods or dietary strategies aimed at maintaining metabolic health.

Diet-induced dysregulation of hepatic lipid metabolism is often accompanied by oxidative stress and cellular damage, which are key considerations in food biochemistry and nutritional physiology. In the present study, the elevated serum levels of ALT, AST, ALP, and LDH in HFD-fed mice indicated hepatocyte membrane integrity loss and liver injury [20]. TFSP intervention significantly reduced the activities of these enzymes, and histopathological examination further confirmed attenuated hepatic lipid accumulation and tissue damage. In addition, oxidative stress induced by high-fat feeding represents a critical aspect of diet-related metabolic disturbance. ROS promote lipid peroxidation, leading to the formation of MDA, a recognized marker of oxidative damage in food and biological systems [21]. TFSP treatment was associated with enhanced activities of key antioxidant enzymes—SOD, GSH-Px, and CAT—and reduced MDA levels in the liver [22]. These results indicate that TFSP, as a food-derived component, may ameliorate diet-induced oxidative stress and enhance hepatic antioxidant capacity, suggesting a potential role in maintaining food-relevant physiological homeostasis.

To further elucidate the lipid-modulating properties of TFSP from a food biochemistry perspective, we examined its effects on the expression of key regulators involved in hepatic lipid metabolism. Our findings reveal that TFSP intervention was associated with altered expression of proteins central to lipogenesis, fatty acid oxidation, and triglyceride hydrolysis. Specifically, TFSP-treated mice exhibited lower levels of ACC and FAS, alongside higher levels of CPT-1α, PPARα and ATGL, compared to the HFD group. These coordinated changes suggest a potential shift toward enhanced mitochondrial β-oxidation and triglyceride hydrolysis, and suppressed de novo lipogenesis [23,24]. The observed modulation of these metabolic pathways provides correlative insight into how this flavonoid-rich fraction might alleviate hepatic lipid accumulation under high-fat dietary conditions. Notably, the upregulation of ATGL, a key lipolytic enzyme, together with its known functional association with PPARα activation, supports the hypothesis that TFSP may promote lipid catabolism [25,26]. However, further studies—such as those using specific inhibitors or gene knockout models—are required to establish direct causal relationships. These findings contribute to our understanding of how flavonoids from food sources may modulate hepatic lipid homeostasis through coordinated changes in multiple metabolic pathways, highlighting their potential application in functional foods aimed at managing diet-induced metabolic alterations.

On the other hand, prolonged HFD exposure can compromise gut microbial diversity [27]. The restoration of community diversity under TFSP intervention is a complex process that likely involves multiple mechanisms. The HFD decreased the species diversity of the intestinal microbiota, increased the relative abundance of *Firmicutes*, and decreased the relative abundance of *Bacteroidetes*, thereby elevating the F/B ratio. Conversely, TFSP treatment was associated with a significant reduction in the F/B ratio. In addition, the relative abundance of *Allobaculum*, *Blautia* and *Faecalibaculum* were significantly higher in the HFD group compared to the NC group, with *Blautia* showing the most substantial increase. Elevated *Allobaculum* levels have been linked to increased risks of hyperlipidemia, hyperglycemia and hepatic steatosis [28]. *Faecalibaculum* is a bacterium that produces short-chain fatty acids (SCFAs), which are among the most crucial substances for maintaining intestinal health. The increased abundance of *Faecalibaculum* in the HFD group may represent an adaptive response, whereby the host recruits this specific bacterial population to mitigate HFD-induced damage through the production of SCFAs. As a key member of the *Verrucomicrobiota*, *Akkermansia* degrades mucins, thereby maintaining the integrity of the host intestinal mucosal barrier [29]. Thus, the increased relative abundance of *Akkermansia* observed in TFSP-treated mice may contribute to mitigating HFD-induced intestinal permeability. In summary, flavonoids may rely on metabolic transformations by gut microbes to exert a range of physiological functions, ultimately contributing positively to human health. It should be noted, however, that these associations do not establish causality, and further mechanistic studies—including fecal microbiota transplantation and metabolomic analyses—are needed to confirm the role of specific microbial taxa in the lipid-lowering effects of TFSP.

This study demonstrates that TFSP administration is associated with amelioration of HFD-induced hyperlipidemia in mice, along with coordinated changes in hepatic lipid metabolism and gut microbiota composition. However, several limitations must be acknowledged. First, the study did not investigate toxicological considerations or the long-term safety of TFSP, which are critical for its practical application as a functional ingredient. Second, the specific flavonoid components responsible for the observed effects remain unidentified, and the mechanisms of individual components have not been elucidated. Third, the gut microbiota analysis was limited to 16S rRNA sequencing, without direct measurement of microbial metabolites (e.g., SCFAs) and their potential link to hepatic lipid metabolism. Future research should include systematic toxicological evaluations, component-specific bioactivity studies, and integrated metabolomic–microbiomic approaches to clarify the causal relationships underlying the hypolipidemic effects of TFSP.

## 5. Conclusions

TFSP intervention markedly suppressed HFD-induced body weight gain in mice and significantly improved serum and hepatic lipid profiles, as evidenced by decreased levels of TC, TG, and LDL-C, along with increased HDL-C. Moreover, TFSP enhanced the activities of key antioxidant enzymes (SOD, GSH-Px, and CAT) and reduced serum levels of ALT, AST, AKP, LDH, and MDA, collectively indicating improved antioxidant capacity and mitigation of HFD-induced hepatic damage. From a food chemistry perspective, these effects underscore the potential of TFSP as a bioactive food ingredient with antioxidant and lipid-modulating properties. Furthermore, TFSP positively influenced the gut microbiota composition by lowering the *Firmicutes/Bacteroidetes* ratio, enriching beneficial genera such as *Akkermansia* and *Faecalibaculum*, and suppressing *Allobaculum*. These findings highlight the role of TFSP in modulating food-relevant physiological pathways through dietary intervention. This study emphasizes the potential of Sea buckthorn pericarp pomace—a food processing by-product—as a source of functional flavonoids for developing lipid-lowering food ingredients, thereby adding value to food industry waste and supporting sustainable food production systems.

## Figures and Tables

**Table 1:** Identification and analysis of TFSP.

Peak Number	Retention Time (min)	Name	Molecular Formula	Concentration (ng/mg)	Categorization
1	0.99	Gallic acid	C_7_H_6_O_5_	3.007 ± 0.446	Phenolic Acids
2	1.91	3,4-Dihydroxybenzoic acid	C_7_H_6_O_4_	2.548 ± 0.578	Phenolic Acids
3	3.19	Protocatechualdehyde	C_7_H_6_O_3_	18.988 ± 0.384	Phenolic Acids
4	3.3	Phthalic acid	C_8_H_6_O_4_	0.601 ± 0.173	Phenolic Acids
5	3.4	4-Hydroxybenzoic acid	C_7_H_6_O_3_	1.33 ± 0.155	Phenolic Acids
6	3.98	Catechin	C_15_H_14_O_6_	994.812 ± 0.845	Flavonoids
7	4.19	Vanillic acid	C_8_H_8_O_4_	21.603 ± 0.628	Phenolic Acids
8	4.34	Caffeic acid	C_9_H_8_O_4_	0.726 ± 0.048	Phenolic Acids
9	4.55	Syringic acid	C_9_H_10_O_5_	7.284 ± 0.162	Phenolic Acids
10	4.81	Epicatechin	C_15_H_14_O_6_	82.49 ± 0.135	Flavonoids
11	4.85	Dihydromyricetin	C_15_H_12_O_8_	22.136 ± 0.422	Flavonoids
12	5.17	Vanillin	C_8_H_8_O_3_	12.488 ± 0.151	Phenolic Acids
13	5.32	p-Hydroxycinnamic Acid	C_9_H_8_O_3_	34.16 ± 0.485	Phenolic Acids
14	5.49	Syringaldehyde	C_9_H_10_O_4_	5.63 ± 0.648	Phenolic Acids
15	5.6	Rutin	C_27_H_30_O_16_	1348.81 ± 0.179	Flavonoids
16	5.6	Salicylic acid	C_7_H_6_O_3_	7.591 ± 0.466	Phenolic Acids
17	5.64	Vitexin	C_21_H_20_O_10_	0.05 ± 0.043	Flavonoids
18	5.68	Trans-Ferulic acid	C_10_H_10_O_4_	12.291 ± 0.686	Phenolic Acids
19	5.71	Sinapic Acid	C_11_H_12_O_5_	22.333 ± 0.56	Phenolic Acids
20	5.75	Quercetin 3-β-D-glucoside	C_21_H_20_O_12_	558.638 ± 0.391	Flavonoids
21	5.78	Luteoloside	C_21_H_20_O_11_	0.132 ± 0.006	Flavonoids
22	5.85	(+)-Dihydroquercetin	C_15_H_12_O_7_	30.906 ± 0.347	Flavonoids
23	5.88	Genistin	C_21_H_20_O_10_	0.364 ± 0.048	Flavonoids
24	6.05	Benzoic acid	C_7_H_6_O_2_	43.597 ± 0.731	Phenolic Acids
25	6.05	Kaempferol-3-O-glucoside	C_21_H_20_O_11_	102.69 ± 0.427	Flavonoids
26	6.44	(+)-Dihydrokaempferol	C_15_H_12_O_6_	2.903 ± 0.029	Flavonoids
27	6.69	Resveratrol	C_14_H_12_O_3_	0.608 ± 0.011	Phenolic Acids
28	6.81	Daidzein	C_15_H_10_O_4_	0.008 ± 0.004	Flavonoids
29	7.05	Luteolin	C_15_H_10_O_6_	0.87 ± 0.016	Flavonoids
30	7.09	Quercetin	C_15_H_10_O_7_	111.289 ± 0.254	Flavonoids
31	7.14	Hydrocinnamic acid	C_9_H_10_O_2_	2.921 ± 0.39	Phenolic Acids
32	7.24	Trans-Cinnamic acid	C_9_H_8_O_2_	4.106 ± 0.44	Phenolic Acids
33	-	Naringenin Chalcone	C_15_H_12_O_5_	-	Flavonoids
34	7.55	Phloretin	C_15_H_14_O_5_	0.089 ± 0.011	Flavonoids
35	7.58	Naringenin	C_15_H_12_O_5_	3.32 ± 0.439	Flavonoids
36	7.58	Apigenin	C_15_H_10_O_5_	0.269 ± 0.014	Flavonoids
37	7.67	Kaempferol	C_15_H_10_O_6_	21.144 ± 0.286	Flavonoids
38	7.73	Isorhamnetin	C_16_H_12_O_7_	182.445 ± 0.62	Flavonoids
39	7.93	Isoliquiritigenin	C_15_H_12_O_4_	0.007 ± 0.001	Flavonoids
40	9.76	Gossypol	C_30_H_30_O_8_	0.344 ± 0.024	Polyphenol

## Data Availability

The original contributions presented in the study are included in the article/Supplementary Material, further inquiries can be directed to the corresponding author.

## Supplementary Materials

The following supporting information can be downloaded at: https://www.mdpi.com/article/10.3390/foods15061049/s1, Figure S1. α-diversity analysis. A. Ace index; B. Chao index; C. Shannon-wiener index; D. Simpson index. *n* = 6 per group. Data were presented as mean ± SD. Compared with the NC group, the HFD group exhibited significant reductions in the Ace, Chao1 and Shannon indices, indicating diminished gut microbial richness and alpha diversity; TFSP intervention partially reversed these deficits. Figure S2. Beta diversity index. A. Non-metric multidimensional scaling analysis; B. Anosim analysis. *n* = 6 per group. NMDS revealed distinct clustering of the HFD group away from the NC group, while TFSP-treated groups showed partial restoration toward the NC group profile. ANOSIM confirmed that between-group differences exceeded within-group variation, supporting the validity of the experimental grouping.

## Abbreviations

The following abbreviations are used in this manuscript:

TFSP	Total flavonoids of *Sea buckthorn* pericarp pomace
HFD	High-fat diet
ACC	Acetyl-CoA carboxylase
FAS	Fatty acid synthase
CPT-1α	Carnitine palmitoyltransferase-1α
ATGL	Adipose triglyceride lipase
PPARα	Peroxisome proliferator-activated receptor alpha
ROS	Reactive oxygen species
SOD	Superoxide dismutase
CAT	Catalase
GSH-Px	Glutathione peroxidase
LDL-C	Low-density lipoprotein cholesterol
HDL-C	High-density lipoprotein cholesterol
TC	Total cholesterol
TG	Triglycerides
XZK	Xuezhikang
F/B	Firmicutes/Bacteroidetes
