# Supplementary material for: Sea Buckthorn Pericarp Flavonoids Improve Diet-Induced Hyperlipidemia via Coordinated Modulation of Hepatic Lipid Metabolism and Gut Microbiota
Source: Foods. 2026 Mar 17;15(6):1049. doi: 10.3390/foods15061049 (PMC13025354; doi:10.3390/foods15061049)
Supplement: Supplementary file 1 [file foods-15-01049-s001.zip › foods-4137785-supplementary.pdf]

Supplementary materials

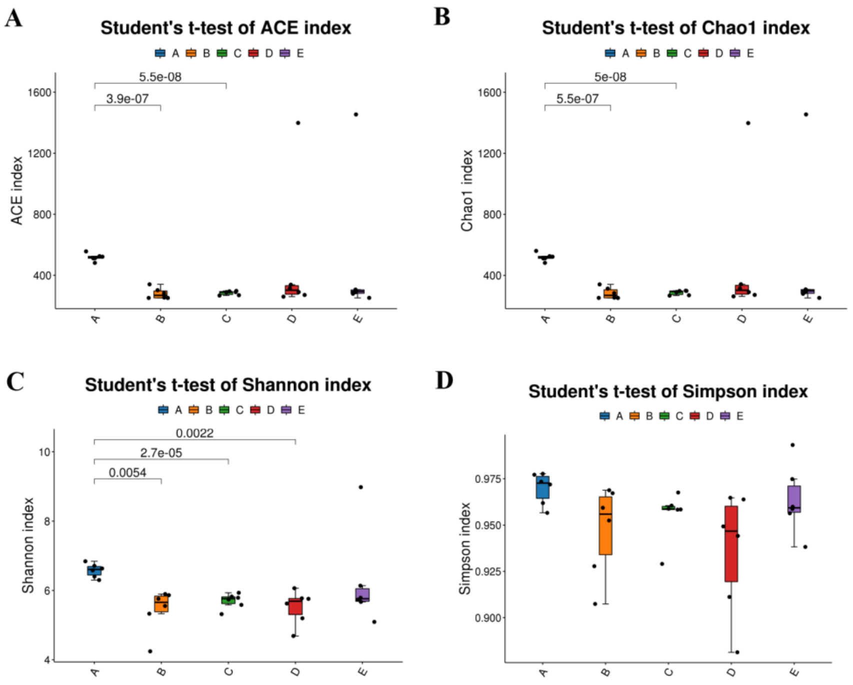

Figure S1. α-diversity analysis. A. Ace index; B. Chao index; C. Shannon-wiener index; D. Simpson index. n = 6 per group. Data were presented as mean ± SD. Compared with the NC group, the HFD group exhibited significant reductions in the Ace, Chao1 and Shannon indices, indicating diminished gut microbial richness and alpha diversity; TFSP intervention partially reversed these deficits.

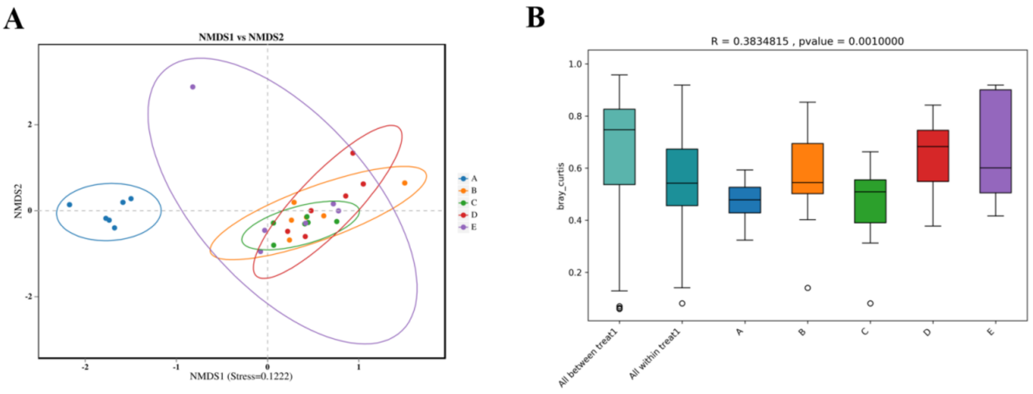

Figure S2. Beta diversity index. A. Non-metric multidimensional scaling analysis; B. Anosim analysis. n = 6 per group. NMDS revealed distinct clustering of the HFD group away from the NC group, while TFSP-treated groups showed partial restoration toward the NC group profile. ANOSIM confirmed that between-group differences exceeded within-group variation, supporting the validity of the experimental grouping.
